# Inflammatory Factors and Exercise in Chronic Kidney Disease

**DOI:** 10.1155/2013/569831

**Published:** 2013-05-13

**Authors:** Maurice Dungey, Katherine L. Hull, Alice C. Smith, James O. Burton, Nicolette C. Bishop

**Affiliations:** ^1^School of Sport, Exercise and Health Sciences, Loughborough University, Leicestershire LE11 3TU, UK; ^2^Leicester Kidney Exercise Team, Leicester General Hospital, Gwendolen Road, Leicester LE5 4PW, UK; ^3^Department of Infection, Immunity and Inflammation, Maurice Shock Medical Sciences Building, University Road, Leicester LE1 9HN, UK

## Abstract

Patients with chronic kidney disease frequently present with chronic elevations in markers of inflammation, a condition that appears to be exacerbated by disease progression and onset of haemodialysis. Systemic inflammation is interlinked with malnutrition and muscle protein wasting and is implicated in a number of morbidities including cardiovascular disease: the most common cause of mortality in this population. Research in the general population and other chronic disease cohorts suggests that an increase in habitual activity levels over a prolonged period may help redress basal increases in systemic inflammation. Furthermore, those populations with the highest baseline levels of systemic inflammation appear to have the greatest improvements from training. On the whole, the activity levels of the chronic kidney disease population reflect a sedentary lifestyle, indicating the potential for increasing physical activity and observing health benefits. This review explores the current literature investigating exercise and inflammatory factors in the chronic kidney disease population and then attempts to explain the contradictory findings and suggests where future research is required.

## 1. Chronic Kidney Disease

Chronic kidney disease (CKD) refers to the progressive and irreversible decline in renal function and is defined as kidney damage for ≥3 months based on findings of abnormal structure or function or glomerular filtration rate (GFR) <60 mL/min/1.73 m^2^ for ≥3 months with or without evidence of kidney damage [1]. There are five different stages of CKD, determined by measuring a patient's estimated GFR (eGFR). Severity of disease is classified using stages of increasing severity from 1 to 5 (Table 1).

Patients with CKD stages 1-2 have relatively normal eGFR and few symptoms. In secondary care, most CKD patients will either be predialysis (CKD stages 3–5) or end-stage renal disease (ESRD) patients. Management of predialysis patients aims to reduce the rate of decline in renal function and the likelihood of reaching ESRD, avoid and treat complications and comorbidities of CKD, and provide symptomatic relief. 

Patients with ESRD require renal replacement therapy (RRT), a treatment which is advised to start once eGFR <15 mL/min and the patient is symptomatic [1]. RRT options include transplantation or dialysis: haemodialysis (HD), haemofiltration, and peritoneal dialysis (PD). Transplantation and HD are the most common types of treatment modalities, accounting for 48% and 44% of RRT techniques respectively [3].

The numerous aetiologies of CKD can be grouped as genetic, glomerular, vascular, and tubulointerstitial diseases or due to urinary tract obstruction [4]. The most common causes are related to diabetes and hypertension [1]. Despite the vast number of causes, classification of renal function into the five stages and long-term management are usually similar [4]. Progressive renal dysfunction is associated with numerous complications as a result of the kidney's regulatory, endocrine, excretory, and metabolic functions. Such complications include anaemia, renal osteodystrophy, pruritus, nephrogenic systemic fibrosis, metabolic abnormalities (e.g., gout, insulin resistance, and dyslipidaemia), endocrine abnormalities (e.g., impaired growth in children due to perturbations in growth hormone action and erectile dysfunction due to decreased testosterone levels), muscle dysfunction, nervous system dysfunction, calciphylaxis, and cardiovascular disease [4]. These complications and the subsequent symptoms may vary between individuals, the origin of the CKD, and comorbidities. However, the majority of complications are exacerbated by declining renal function and progression to ESRD.

CKD is a worldwide health problem which increases in prevalence with age; a global systematic review reported a median prevalence of CKD stage 3 and greater of 7.2% in individuals aged over 30 years of age, and between 23.4% and 35.8% in those aged 64 years or older [5]. The risk of mortality is substantially elevated in this population [6]; the average 5-year survival of western dialysis patients aged 60 years is approximately 45% (data from UK, Swedish, and USA registries).

Not all CKD patients progress to ESRD; rather, in all stages of CKD, death is a far more common event than the initiation of RRT. Keith et al. followed 28,000 patients with CKD stages 2, 3, and 4 with 1.1%, 1.3%, and 19.9% reaching ESRD and mortality rates of 19.5%, 24.3%, and 45.7% respectively [7]. Furthermore, in a retrospective cohort study observing 1076 patients over a five- and a half-year period found that 4% developed ESRD and 69% had died, with 46% of deaths cardiovascular in origin [8]. Cardiovascular disease (CVD) is the most frequent cause of mortality, with CKD itself being an established risk factor for CVD [9]. Deteriorating kidney function is associated with an increased risk of CVD at every stage of CKD [10]. 

As the major cause of comorbidity and mortality in the CKD population, cardiovascular risk is an important target for intervention. The development of coronary atherosclerosis and endothelial dysfunction is a multifaceted inflammatory process [4]. In CVD, the presence of inflammation has been implicated at inception and every stage of the atherosclerotic process thereafter: from adhesion molecule expression on endothelial cells, penetration of monocytes into the intima, creation of fatty streaks, enhanced inflammatory response, development of the complex plaque, weakening of the fibrous collagen cap, and increased risk of thrombosis [11]. Chronic inflammation is also associated with other complications and comorbidities frequently seen in CKD such as conditions affecting the endocrine system (insulin resistance and metabolic syndrome) and neurological system (depression) [12]. CKD itself presents a complex multidimensional inflammatory condition. 

## 2. Inflammation and Chronic Kidney Disease

Inflammation is a rapid and acute protective response to infection or trauma. The activation of the complement pathway stimulates the degranulation of mast cells and the release of inflammatory cytokines [4]. This has both local (redness, swelling, heat production, and pain) and systemic consequences (fever) due to changes in local blood flow and the effect of cytokines on the hypothalamus, respectively. Systemic inflammatory cytokines (Interleukin-1 (IL-1), IL-6, and tumour necrosis factor-alpha (TNF-*α*)) stimulate hepatocytes to secrete the acute-phase protein C-reactive protein (CRP), the most widely used marker of inflammation. The initial reaction to damaging stimuli is a normal and necessary process to help contain infection and begin the cascade of events involved in the immune response. However, when the initial insult cannot be acutely resolved or when anti-inflammatory systems responsible for regulating inflammation are dysfunctional, inflammation persists. A chronic inflammatory state is harmful, rather than protective, as it may result in end organ and vascular damage [4]. 

In CKD, chronic inflammation plays an important role in the disease process and high levels of CRP appear to accompany reduced renal function [13]. Within the predialysis CKD population the prevalence of inflammation is great and is an important indicator of patient health and outcome; high levels of CRP reflect a chronic inflammatory state associated with reduced serum albumin levels, inadequate response to erythropoietin replacement, and greater hospitalisation [14]. However, the actual effect of chronic inflammation on renal function is unclear as although inflammation is prevalent within the predialysis CKD population, the relationship between the level of inflammation (using CRP or inflammatory cytokines such as IL-6 as surrogate markers), and eGFR has not been found to correlate [14, 15], as may be expected. In addition, in many of the aetiologies of CKD, inflammation has a role in their pathogenesis. Thus, it is unclear whether it is renal insufficiency or the disease origin causing chronic inflammation in the first stages of CKD.

In ESRD, the process of HD itself may contribute to the pro-inflammatory state. For instance, exposure to the dialysis membrane may stimulate an acute-phase response and an increase in inflammatory cytokine levels [16]. Furthermore, it appears that this exposure and the type of dialysis membrane itself are greater determinant in stimulating the acute-phase response than the bacterial nature of the dialysate [17]. However, HD is not the only source of inflammation since predialysis CKD patients have elevated levels of inflammatory markers, as previously discussed, and some research has found a number of HD patients that have normal CRP levels [18]. Thus, it may be proposed that other mechanisms are involved in the inflammatory state, including processes contributing to uraemia [19]. A number of factors with the potential to induce inflammation in CKD have been described (Table 2). 

While the source(s) of chronic inflammation in CKD can vary, the negative implications of elevated inflammatory markers are clear. For example, an increase or persistent elevation in markers of inflammation (CRP, IL-6, and TNF-*α*) are highly predictive of mortality [20].

The relatively long 19-hour half-life of CRP makes it easy to detect; thus, CRP is frequently used as a clinical marker of inflammation. However, IL-6 has been reported as a better prognostic marker than both CRP and TNF-*α* [21] in haemodialysis and predialysis CKD populations [22]. Moreover, IL-6 is a particularly interesting molecule due to its divergent roles as both a pro- and anti-inflammatory factor.

The role of IL-6 appears to be context and source dependent. At rest, circulating concentrations of IL-6 are attributed to adipocytes and macrophages resident within adipose tissue [23], uraemia-induced immune dysfunction [24], and other causes (Table 2). An acute infusion of IL-6 leads to transient increases in a number of anti-inflammatory factors (cortisol, IL-10, IL-1 receptor antagonist (IL-1ra)) without a change in TNF-*α* [25]; the anti-inflammatory effect of myocyte secretion of IL-6 is discussed later. Thus, it appears that transient increases in IL-6 which are well-regulated by an effective anti-inflammatory response are not detrimental; it is when IL-6 is chronically elevated and represents an inflammatory milieu (e.g., immune dysfunction) that IL-6 concentrations are associated with poor outcome.

Pro-inflammatory cytokines are pleiotropic in their nature and impact upon numerous conditions that frequently accompany CKD. Systemic inflammation has a role in malnutrition and protein-energy wasting, atherosclerosis, endocrine disorders, and depression [27]. With regards to malnutrition and protein-energy wasting in CKD, these comorbidities are interlinked and share common aetiologies, yet the contribution of each aspect to poor outcome is not well defined. Therefore, “malnutrition-inflammation-cachexia syndrome” (MICS) has been suggested to denote the important contribution of these conditions to outcome in CKD [28]. The adverse effects of MICS develop at a greater rate in ESRD than the traditional risk factors for CVD, such as obesity and hypercholesterolaemia [29], and are important predictors of prognosis [30]. 

IL-6 has been implicated in the breakdown of muscle protein in other cachexic populations [31] and related to markers of wasting in CKD patients [32]. IL-6 inhibits insulin-like growth factor-1 (IGF-1) secretion and consequently growth and repair [33]. Insulin resistance, reduced appetite, increased basal energy expenditure, and activation of the ATP-ubiquitin proteolytic pathway have been suggested as conditions by which chronic inflammation instigates muscle wasting [27]. 

Chronic elevations in leptin may also contribute to muscle protein wasting [34]. A leptin receptor-deficient mice model exhibited elevated leptin levels after undergoing nephrectomy but resisted cachexic effects [35].

It is noteworthy that a number of anti-inflammatory cytokines are also elevated in CKD. For instance, IL-10 increases in response to inflammation and in healthy individuals it is predominantly cleared by the kidneys. Consequently IL-10 is frequently found to be higher in CKD than healthy populations [36]. In addition to its anti-inflammatory benefits, IL-10 may have anti-atherosclerotic effects [36]. In the majority of CKD patients the elevations in pro-inflammatory factors outweigh any anti-inflammatory increases; however, the subjects with the highest IL-10 levels have a better immune balance, as shown by improved response to vaccination [37]. 

Overall, CKD patients exhibit elevations in markers of chronic inflammation [26]. Since inflammation, malnutrition and protein-energy wasting are significant contributors to mortality in CKD patients [38], any treatments which may positively influence these conditions should be explored. The concept of exercise as medicine is an area of increasing interest due to its wide range of diverse beneficial effects [39]. 

## 3. Physical Activity and Inflammation in the General Population

There is now a growing body of evidence to support the notion that circulating markers of systemic inflammation are lower in individuals who regularly engage in physical activity. Indeed, a systematic review of literature on this topic published between 1975 and 2004 concluded that regular physical activity is associated with a long-term “anti-inflammatory effect” [40]. For example, cross-sectional observational studies in apparently healthy people consistently report inverse relationships between circulating markers of systemic inflammation and physical activity levels or fitness [41–44]. Key criticisms of this literature have been the tendency to focus on cohorts of a narrow age range (often middle-aged or older), not taking into account confounders such as BMI and leisure time activity as opposed to overt exercise activities. A recent study to address these points assessed CRP and several cytokines including IL-6, IL-10, TNF-*α*, and monocyte chemoattractant protein-1 (MCP-1) in almost 1000 men and women (40% men, 60% women) ranging in age from 18 to 85 years and BMI of 16.7–52.7 kg/m^2^ [45]. Plasma levels of CRP, IL-6, and TNF-*α* were significantly lower when comparing high-to-low tertiles for leisure time exercise frequency and perceived levels of fitness, even after adjustment for BMI, age, sex, and smoking. Interestingly, despite this effect BMI was still ranked as the most important influencing factor for CRP and IL-6 levels, with age the most important for TNF-*α* levels. There was no effect of either perceived fitness or leisure time exercise frequency on IL-10 and MCP-1. 

The findings of a recent 10-year follow-up study also lend strong support to the role of regular exercise in lowering plasma levels of IL-6 and CRP [46]. Findings from a cohort of over 4000 male and female UK Government workers found that those meeting the physical activity recommendations for cardiovascular health (2.5 h/wk of moderate to vigorous physical activity) had lower CRP and IL-6 levels at baseline and at 10-year follow-up, even after adjusting for age, sex, smoking, BMI, employment grade and chronic illness. An increase in physical activity was also associated with lower levels of IL-6 and CRP at follow-up. Importantly, these observed associations were independent of central adiposity as the effects persisted when data were adjusted for waist circumference. 

The findings of Hamer et al. [46] appear to suggest that regular activity needs to be performed for a period of years before the benefits of exercise independent of weight loss and reduced adiposity are observed. Certainly the majority of intervention studies that have implemented aerobic training programmes lasting 12 months or less generally report either no effect or a lowering of inflammatory markers only when in combination with weight loss [47–50]. However, differences in exercise prescription, baseline levels of inflammatory markers, and time after intervention of the final blood sample may also influence study findings: Thompson et al. reported significant, albeit modest (0.4 pg/mL) falls in plasma IL-6 in previously sedentary men after just 12 weeks of a 24-week progressive training programme (progressing from 30-minute sessions at 50% of maximal aerobic capacity to 1-hour sessions at 70% of maximal aerobic capacity), compared with non-exercising controls [50]. This was associated with significant, yet modest falls in body mass (~1.6 kg) and BMI (~0.7 kg/m^2^) after 24 weeks. However, the effect on IL-6 (but not body mass or BMI) was reversed within 2 weeks of the last training session; this could have important implications because it is not uncommon for studies to collect post-intervention blood samples 7–14 days after the last training session. 

Examination of the literature suggests that investigation of the mechanisms by which exercise exerts its anti-inflammatory effects has been largely focused on the effects of reduced adiposity and expression and release of adipose tissue-derived inflammatory cytokines [51]. Adipose tissue is recognised as a metabolically active tissue that plays a key role in the development of chronic low-grade inflammation. Adipose tissue is able to produce inflammatory cytokines such as TNF-*α*, IL-1*β*, IL-6, and several potent chemoattractant cytokines (chemokines) including MCP-1, macrophage inflammatory protein-1a (MIP-1a) and the T (and monocyte) chemokine regulated on activation, normal T cell secreted, and expressed (RANTES) [52, 53]. The accumulation of monocytes as macrophages in adipose tissue is thought to be a major source of increased systemic concentrations of inflammatory cytokines [54]. With this in mind, increased physical activity and dietary restriction that results in a negative energy balance and consequently reduces adiposity, have been typically suggested as the main mechanism by which exercise exerts its beneficial effects on the level of circulating inflammatory markers. However, non-obese healthy individuals who take part in regular physical activity also have reduced circulating levels of systemic inflammation, particularly IL-6 and CRP, compared with non-obese healthy individuals who adopt a sedentary lifestyle [41, 42, 55]. Furthermore, the ten-year observational study by Hamer et al. reported lower levels of IL-6 and CRP in those meeting the UK Physical Activity Guidelines for health compared with those that did not, and this effect persisted even after adjusting for body mass index and waist circumference [46]. Therefore the positive impacts of increasing activity levels are not restricted to those achieved through reducing adiposity.

Several other potential mechanisms have now been suggested to contribute to the lowering of circulating markers of systemic inflammation in those who are physically active [51]. One possible mechanism that has been the focus of much recent attention is the increased production and release of cytokines from contracting skeletal muscle “myokines” during exercise [56]. Muscle and circulating levels of IL-6 increase in response to exercise loads of sufficient duration and to a lesser extent, intensity. Although increases in circulating IL-6 levels of over 100-fold have been detected after prolonged exercise lasting over 2.5 hours [57], more modest increases are detectable after shorter-duration exercise [58] and also in response to resistance exercise [59, 60]. A major stimulus for IL-6 release is a fall in muscle glycogen content [61, 62] but increases in intracellular calcium levels, and formation of reactive oxygen species also plays a role in activating transcription factors known to regulate synthesis of IL-6 [58]. Increases in circulating levels of IL-6 appear to stimulate the release of anti-inflammatory cytokines IL-10 and IL-1ra and inhibits the release of the pro-inflammatory cytokines IL-1*β* and TNF-*α* [56], creating a circulating “anti-inflammatory” environment with every bout of exercise. It should also be noted that the half-life of IL-6 is prolonged by combining with the soluble IL-6 receptor (sIL-6R), muscle membrane expression of sIL-6R is also increased by exercise training [61].

Muscle release of IL-6 and the subsequent cascade of anti-inflammatory cytokines cannot be the sole mechanism underlying the so-called anti-inflammatory effects of exercise because short durations of low-to-moderate intensity of exercise are associated with reductions in circulating concentrations of markers of inflammation and yet do not stimulate IL-6 release [58]. A recent review of further mechanisms thought to be involved in the beneficial effects of exercise on inflammation [51] highlighted reduced expression of toll-like receptors on monocytes and macrophage with subsequent inhibitory effects on monocyte/macrophage IL-6 and TNF-*α* production, inhibition of monocyte/macrophage infiltration into adipose tissue, phenotypic switching of macrophages from a pro-inflammatory phenotype to an anti-inflammatory phenotype within adipose tissue, a reduction in the circulating numbers of pro-inflammatory monocytes, and an increase in the circulating numbers of regulatory T cells as having a potential role. In particular, toll-like receptors (TLRs) may play a key role in the link between a sedentary lifestyle, inflammation, and disease because both exercise training studies and cross-sectional comparisons between physically active, and sedentary individuals have shown reduced monocyte TLR4 expression and stimulated IL-6 release with increased amounts of activity [63, 64].

## 4. Physical Activity and Chronic Kidney Disease

Physical inactivity is associated with an increased risk of development of CKD [65, 66]. Inactive ESRD patients are at an increased risk of mortality (hazard ratio: 1.62) [67] and patients that report exercising 2-3 or 4-5 times a week have a reduced relative risk of mortality (0.74 and 0.70 resp.) in comparison to sedentary counterparts [68].

As a whole CKD patients are a sedentary population, activity levels are significantly lower than those recommended by national guidelines [69]. Peak aerobic capacity is markedly lower in CKD stages 3–5 than healthy reference groups [70]. Consequently, results from physical function tests (sit-to-stand test, hand-grip, up-and-go test, and 6-minute walk test) are frequently lower than would be expected [71–73]. 

Maximal exercise tolerance is affected in the very early stages of CKD [70]. Exercise capacity and physical activity levels correlate with eGFR [74, 75] therefore suggesting that physical fitness and activity levels decline as the disease severity worsens. ESRD patients have significantly lower daily activity-related energy expenditure than healthy sedentary controls, and this disparity is exacerbated on days when patients receive haemodialysis treatment [69, 76]. Self-reported physical activity levels are a good predictor of post-dialysis fatigue scores; patients who are most active report reduced fatigue levels [77]. Consequently, decreased physical activity levels and fitness represent the beginning of a vicious cycle of declining fitness, activity, physical function, and disability which if left unchecked will continue to escalate. A shift in sedentary behaviour to a more active lifestyle should have survival benefits in individuals with CKD.

## 5. Exercise and Inflammatory Factors in Chronic Kidney Disease

### 5.1. Observational Studies

Despite smaller cohorts and a limited depth of literature, associations between inflammatory markers and physical fitness or activity levels in CKD patients correspond with findings from healthy populations. In a study of over 200 dialysis patients self-reported physical activity levels correlated inversely with CRP [78]. These findings were supported in ESRD patients who wore SenseWear physical activity monitors for 7 days; circulating CRP >5 mg/L was associated with lower energy expenditure and steps walked than patients with lower concentrations of CRP [79]. In contrast Zamojska and colleagues found no such relationships between CRP and steps walked [80]; this may be explained by the short duration of activity monitoring (2 days) and the exclusion of all patients who reported difficulty walking or physical impairments.

In a mixed predialysis and ESRD patient cohort the main determinant of CRP was VO_2peak_; the higher the aerobic capacity of the patient, the lower their inflammatory state (*R* = −0.51) [81]. Large cohort studies measuring numerous inflammatory factors are required to further establish the relationship between greater fitness and activity levels and an improved lower systemic inflammatory state. 

Observational studies do not give an insight into the mechanisms behind relationships, nor do they help provide evidence of causation. Although there is an association between activity levels and inflammatory factors, it is unclear as to whether this is due to a protective role of regular physical activity. This observed association may be attributed to the role of inflammatory cytokines in muscle catabolism [27], and subsequent reduction in physical capacity and physical activity levels. However, since numerous training studies have been shown to yield favourable adaptations to aerobic and functional capacity [82], a two-way negative relationship between physical activity and inflammation is likely.

### 5.2. Longitudinal Studies

A growing number of exercise interventions have been trialled in CKD patients, a recent review and meta-analysis concluded that regular exercise in the CKD population provides significant improvements in physical fitness, cardiovascular dimensions, nutritional parameters, and health-related quality of life [83]. Studies reporting inflammatory factors as outcome measures are scarce. 

#### 5.2.1. ESRD Patients

A few early exercise intervention studies investigating inflammatory factors within ESRD patients have been published in the last decade; to date results have been inconclusive (Table 3). A few studies have reported no changes in inflammatory cytokines that is, IL-6 [84, 85]and the acute phase protein CRP [86–89], and others have reported decreased levels of circulating CRP after training [90–94].

As an illustration of the contradictory findings: Cheema and colleagues reported an improvement in inflammatory status due to a reduction in CRP concentrations after 12 weeks of progressive resistance training [92]. However, upon later analysis no changes in pro- or anti-inflammatory cytokines were reported in the same cohort (IL-1*β*, TNF-*α*, IL-6, IL-8, IL-10, and IL-12) although greatest muscular hypertrophic adaptations were associated with the greatest reductions in resting IL-6 levels [85]. 

Notably, two studies from a single research group gave surprisingly large positive changes after a modest training programme. In two randomised controlled trails, 8 weeks of cycling for 10–30 minutes at intensity perceived to be “somewhat hard” produced over 80% reductions in circulating CRP and a 20% decline in serum leptin concentrations [93, 94]. Since other studies have not found such dramatic results in longer studies of patients training at a greater intensity, it appears advisable to interpret these results with caution.

#### 5.2.2. Predialysis Patients

There is an even greater paucity of research in predialysis patients (Table 4). Exercise research is in its infancy in this disease population, only a few relevant studies have been completed which report conflicting results. A 12-week supervised progressive resistance training programme produced type I and type II muscle fibre hypertrophy, improvements in muscular strength, and concomitant reductions in IL-6 and CRP [96]. Muscle fibre cross-sectional area (CSA) hypertrophy and improvements in muscular strength were inversely associated with changes in circulating IL-6. This is in agreement with resistance training in HD patients [85]. Whether the associations between reductions in inflammation and improved muscle anabolism are causal is unknown; nevertheless, reductions in resting IL-6 concentrations may facilitate hypertrophy. As discussed earlier, elevations in IL-6 concentrations inhibit insulin-like growth factor secretion, and inflammation is implicated in muscle catabolism [99]. On the other hand, the increased volume of metabolically active muscle may be conducive in reducing IL-6 concentrations at rest.

Elsewhere, a couple of training studies found no alterations in IL-6 or CRP in predialysis patients [97, 98]. Despite a large duration, a programme of supervised mixed aerobic and resistance training lasting 48 weeks improved aerobic capacity but did not alter concentrations of IL-6 or CRP [97]. Unpublished work from our group found that six months of regular walking exercise in predialysis patients reduced IL-6 and increased IL-10 concentrations at rest, therefore improving the ratio of IL-6 to IL-10 toward a less inflammatory environment.

## 6. Weaknesses of Current Literature

Overall, the current available literature is unable to provide conclusive evidence of an anti-inflammatory adaptation to physical training in CKD patients. A number of factors may account for the inconsistent findings reported in intervention studies.

Unfortunately, much of the available research is primitive pilot or feasibility work for which circulating inflammatory factors are secondary outcome measures. Consequently, a number of studies are without a control group to make valid comparisons [89–91]. 

None of the present available research has an exercise group greater than 30 patients; the small cohorts are perhaps underpowered to preclude type II errors. Large inter-individual differences are seen in IL-6 and CRP in healthy populations [100]; this is exacerbated by the variability of the condition of patients with CKD, and their different comorbidities and aetiology [101]. It is unsurprising that small studies with cohorts of mixed condition have failed to find significant changes. Large-scale multicentre studies are needed with large randomised training cohorts and matched control groups.

There are no set guidelines or exercise protocols for CKD patients, and so a large variety of exercise treatments have been tested. For many patients heart rate and VO_2_ are not appropriate measures of intensity due to medications and haemodialysis treatment; therefore, subjective measures such as the rating of perceived exertion (RPE) are utilised [102]. Differences in exercise modalities, duration and intensity give different training adaptations and therefore make comparisons between separate studies difficult. One study has made the comparison between different exercise modalities in ESRD patients but found no changes in CRP, TNF-*α* or IL-6 in groups performing aerobic exercise, resistance exercise or a combination of the two [86].

Other potential explanations for discrepancies between studies include large variability in baseline inflammatory values of study participants, different assays used, and differences in the time taken to collect post-training samples [50]. 

## 7. Discussion

Data from intervention studies in CKD patients has so far been inconclusive; nonetheless, research in other clinical populations associated with elevated chronic inflammation has shown more promising results. Significant improvements in inflammatory status after short-duration exercise programmes have been seen in patients with type 2 diabetes mellitus [103], metabolic syndrome [104], ischaemic heart disease [105], and chronic heart failure [106]. Why these populations have more frequently reported benefits from exercise than CKD patients is unclear. Wilund and colleagues suggest the anti-inflammatory effects of regular physical activity that are observed in the general population, and other chronic diseases may not be adequate to alter the severe inflammation seen in ESRD [88]. However, it has been reported elsewhere that individuals with the poorest baseline values are often the most susceptible to improvement [50, 105].

The proposed benefits of exercise on inflammation may be a result of repeated acute-bout effects rather than a sustained basal state alteration. The release of the “myokine” IL-6 from contracting muscle stimulates an anti-inflammatory cascade [107]. Basal levels of IL-6, TNF-*α*, and IL-10 are frequently elevated in CKD, especially in ESRD [36]; therefore regular exercise and repeated transient large-scale increases in IL-6 and anti-inflammatory factors that follow (IL-1ra, cortisol and IL-10) may have particularly important anti-inflammatory effects. 

To our knowledge no studies have reported the acute effects of exercise on inflammatory markers in CKD. Unpublished results from our group found an increase in IL-6 and IL-10 concentrations immediately after and 1 hour after an acute bout of 30-minute moderate-intensity walking exercise in predialysis patients; an exercise intensity lower than normally expected to induce IL-6 secretion from muscle [58]. The exercise stimulus required to elicit acute IL-6 secretion from myocytes may in fact be lower in CKD. At rest muscle IL-6 expression is upregulated in ESRD patients displaying an inflammatory response, potentially due to inhibited muscular glucose uptake or increased reactive oxygen species and metabolic acidosis [108], suggesting that a reduced stimulus would be required to expand secretion. In addition, IL-6 release at rest was associated with a negative protein balance in ESRD but not in predialysis CKD patients. Importantly, none of the available literature indicates that exercise may increase basal levels of inflammation or muscle catabolism, signifying a differentiation between IL-6 concentrations at rest and transient elevations due to exercise. Further studies are required to determine the acute IL-6 response to exercise in CKD patients and the duration of such changes. The challenge is in developing exercise programmes which stimulate anti-inflammatory responses but are also feasible and practical in patients with reduced physical function.

Alternatively, it could be suggested that the training stimulus in the current literature is not sufficient to cause an adaptation. In a high-intensity interval training study (3-weekly sessions of 15× 1-minute intervals at 90% HR_peak_) in renal transplant patients large reductions in IL-6 were reported after 10 weeks (before 2.8 ± 0.6 versus after 1.7 ± 0.5 pg/mL) [109]. Although this study had no control group this may represent a training load required to stimulate a significant adaptation.

A reduction in adipose tissue has been proposed as a major mechanism for restricting chronic inflammation [51]. Physical activity has a key role in weight loss programmes within the CKD population; patients who regularly take part in exercise are more likely to succeed in achieving planned weight loss by inducing a greater energy deficit [110]. No studies thus far have investigated inflammatory markers in CKD patients taking part in planned weight-loss programmes. Since adipose tissue and indwelling macrophages are major contributors to circulating concentrations of pro-inflammatory adipokines leptin, resistin, TNF-*α*, and IL-6 amongst others [23], and truncal fat mass is associated with increased levels of inflammation in ESRD [111], it is intuitive to suggest a successful reduction in fat-mass and shift in body composition would have anti-inflammatory benefits in CKD patients; further research is required in this area.

ESRD patients have been reported to have over 3-fold elevations in pro-inflammatory monocytes (CD14^low^CD16^+^) which contribute significantly to inflammation despite representing only 5.5% of monocytes in healthy subjects [112, 113]. Furthermore, TLR2 and TLR4 have been shown to be up-regulated [114], and regulatory T cell numbers and capacity are suppressed [115, 116]. Since exercise training may redress these imbalances in other populations [51], future research should investigate these mechanisms. 

## 8. Conclusions

From the sparse data available some tentative conclusions can be drawn. Observational data suggests that habitual physical activity levels and fitness are associated with a reduced inflammatory profile and consequently improved survival. 

A few, small, short-duration intervention studies which increase physical fitness, strength, and activity levels have mixed effects on systemic inflammation in CKD patients; this follows similar results from short-duration studies in the general population although training in other chronic disease cohorts has demonstrated beneficial effects. In CKD patients there are other numerous benefits of regular exercise, and importantly, it has not been shown to have any detrimental effect in terms of inducing inflammation or muscle catabolism. In fact, the greatest degrees of muscle hypertrophy are associated with the greatest improvements in markers of inflammation. 

Data in healthy groups have shown improvements in inflammatory factors through reductions in fat mass, the regular release of anti-inflammatory myokines, and adaptations to leukocytes including phenotypic switching, expression of TLRs and numbers of circulating regulatory T lymphocytes. These represent future areas for research. 

Development of exercise programmes in chronic kidney disease to maximise benefits for the patient provides a challenge. The CKD population are frequently sedentary and present chronic systemic inflammation; there may be great potential for long-term exercise interventions to shift habitual activity and improve health status. 

## Figures and Tables

**Table 1:** Progression of chronic kidney disease [2].

CKD stage	Description	GFR (mL/min/1.73 m^2^)
1	Renal damage with increased or normal GFR	≥90
2	Renal damage with a mild reduction in GFR	60–89
3a	Moderate reduction in GFR with or without evidence of other renal damage	45–59
3b		30–44
4	Severe reduction in GFR with or without evidence of renal damage	15–29
5	Established renal failure	<15

CKD: chronic kidney disease; GFR: glomerular filtration rate.

Use the suffix (p) to denote the presence of proteinuria when staging CKD and define proteinuria as urinary ACR (albumin : creatinine ratio) ≥30 mg/mmol, or PCR (protein : creatinine ratio) ≥50 mg/mmol.

**Table 2:** Summary of the potential causes of chronic inflammation in chronic kidney disease (adapted from Cheung et al. [26]).

Causes of inflammation in CKD	Additional inflammatory factors relating to dialysis
(i) Decreased GFR (ii) Decreased clearance or increased production of pro-inflammatory cytokines (iii) Volume overload (iv) Oxidative stress (v) Carbonyl stress (vi) Deteriorating nutritional state and food intake (vii) Alterations in body composition (viii) Uraemic toxins (ix) Infection (x) Genetic and epigenetic factors	(i) Intravenous catheter, peritoneal dialysis catheter and its related infections (ii) Dialysis membranes with poor biocompatibility (iii) Impurities in dialysis water and dialysate (iv) Back-filtration or back-diffusion of contaminants (v) Constant exposure to peritoneal dialysis solution (vi) Peritonitis

CKD: chronic kidney disease; GFR: glomerular filtration rate.

**Table 3:** Exercise intervention studies in haemodialysis patients.

Study	Design (Number of patients)	Training	Outcome measures: *Exercise versus control (unless stated otherwise) *
Afshar et al., 2010 [93]	RCT (7 aerobic, 7 resistance, 7 CON)	Aerobic: ID cycling: RPE 12–14 10–30 min, 3x/wk, 8 wks Resistance exercises: RPE 15–17; 3x/wk, 8 wks	Aerobic versus resistance versus CON Hs-CRP: −83.9% versus −67.9% versus +1.5% Serum creatinine: −65.6% versus −59.9% versus +1.2% Albumin: NC

Afshar et al., 2011 [94]	RCT (14 EX versus 14 CON)	ID cycling @ RPE 12–14 10–30 min 3x/wk, 8 wks	Serum leptin: −19.9% versus +29.2% CRP: −83.2% versus +1.2%

Cheema et al., 2007 [92] Cheema et al., 2011 [85]	RCT (24 EX versus 25 CON)	ID PRT: 2 sets 10 exercises @ RPE 15–17 using free weights. 3x/wk, 12 wks	Total strength: +15.2 versus −2.4 kg Mid-thigh circumference: +0.7 versus −0.3 cm Log CRP: −0.08 versus +0.24 TNF-*α*: NC IL-1*β*: NC IL-6: NC IL-10: NC IL-12: NC

Daniilidis et al., 2004 [84]	RCT (20 EX versus 14 CON)	NDT aerobic interval exercises (steps, treadmill, gymnastics, swimming, and ball games) @ 75–85% HR_peak_ 60 min, 3x/wk, 6 months	VO_2peak_: +42% versus NC IL-2: NC IL-4: NC IL-6: NC T-lymphocyte subsets: NC

Golebiowski et al., 2012 [89]	Uncontrolled (29 EX)	ID cycling 3x/wk 3 months	6 min walk velocity: +4% CRP: NC IL-6: NC

Kopple et al., 2007 [86]	RCT (10 end-EX, 15 Str-EX, 12 Com-EX, 14 CON, 20 healthy-CON)	End-EX: ID cycling up to 40 min @ approx. 50% VO_2peak_ Str-EX: NDT leg resistance exercise 3 sets of 6–8 reps @ 80% of 5 RM Com-EX: half End-EX/half Str-EX All: 3x/wk, 18 wks	Duration and work rate of exercise sessions: significant improvement in all exercise groups. CRP: NC TNF-*α*: NC IL-6: NC

Nindl et al., 2004 [91] (Headley et al., 2002 [95])	Uncontrolled (10 EX)	NDT: PRT using 9 resistance machine exercises 1–3 sets 15x reps 2x/wk, 12 wks	6 min walk test distance: +5% Peak torque: +12.6% CRP: week −6: 12.42 ± 2.96 mg/L; week 0: 10.37 ± 2.71 mg/L; week 6: 7.55 ± 1.57 mg/L; week 12: 6.12 ± 1.07 mg/L

Toussaint et al., 2008 [87]	Randomised crossover (*N* = 10 + 9)	ID cycling 30 min 3x/wk 3 months (1 month washout)	3 months EX versus 3 months non-EX CRP: NC

Wilund et al., 2010 [88]	RCT (8 EX versus 9 CON)	ID cycling, 45 min @ RPE 12–14 3x/wk, 4 months	Shuttle walk test distance: +17% versus NC CRP: NC IL-6: NC

Zatuska et al., 2002 [90]	Uncontrolled (10 EX)	ID cycling 30 min, 3x/wk, 6 months	CRP: decrease (*P* < 0.046)

CON: control; CRP: C-reactive protein; EX: exercise; HR_peak_: peak heart-rate; Hs: high-sensitivity; ID: intradialytic; IL: interleukin; NC: no significant changes; NDT: non-dialysis time; PRT: progressive resistance training; RCT: randomised controlled trial; RM: repetition maximum; RPE: rating of perceived exertion; TNF-*α*: tumour necrosis factor-alpha.

**Table 4:** Exercise intervention studies in predialysis chronic kidney disease patients.

Study	Design (Number of patients)	Training	Outcome measures: *exercise versus control (unless stated otherwise) *
Castaneda et al., 2004 [96]	RCT (14 EX versus 12 CON) (median GFR 27.5 ml/min/1.73 m^2^)	Supervised 5x resistance exercise machines 3 sets, 8 reps @ 80% 1 RM (progressive) 3x/wk, 12 wks	Type I muscle fibre CSA: 24% versus −14% Type II muscle fibre CSA: 22% versus −13% Muscle strength: +86 ± 45 kg versus −35 ± 62 kg CRP: −1.7 mg/L versus +1.5 mg/L IL-6: −4.2 pg/mL versus 2.3 pg/mL

Headley et al., 2012 [97]	RCT (10 EX versus 11 CON) (CKD stage 2–4)	Mixed aerobic training up to 55 min @ 50–60% VO_2peak_ and some resistance exercises 3x/wk, 48 wks	VO_2peak_: 18.1 to 19.5 versus 18.8 to 17.0 mL/kg/min hs-CRP: NC IL-6: NC

Leehey et al., 2009 [98]	RCT (7 EX versus 4 CON) [CKD stage 2–4]	Walking exercise >30 min 3x/wk 6 wks supervised followed by 18 wks home based	Exercise duration at 0, 6, and 24 wks: EX: 6.6 to 11.3 to 10.2 min versus CON: NC CRP: NC

CON: control; CRP: C-reactive protein; CSA: cross-sectional area; EX: exercise; GFR: glomerular filtration rate; IL: interleukin; NC: no significant changes; RCT: randomised controlled trial; RM: repetition maximum.
